# A Case Report and Review: Extreme Uterine Torsion Complicated by Couvelaire Uterus and Intrauterine Fetal Demise

**DOI:** 10.1155/crog/8786733

**Published:** 2026-05-15

**Authors:** Eiman Alazemi

**Affiliations:** ^1^ Department of Obstetrics and Gynecology, Maternity Hospital Kuwait, Ministry of Health, Kuwait City, Kuwait, behdasht.gov.ir

**Keywords:** couvelaire uterus, fertility preservation, intrauterine fetal demise, obstetric emergency, uterine torsion

## Abstract

Uterine torsion is an extremely rare obstetric emergency with potentially severe maternal and fetal outcomes. We describe the case of a 36‐year‐old multiparous woman who arrived with sudden, severe abdominal pain and hemodynamic instability. Ultrasound confirmed intrauterine fetal demise (IUFD). Emergency laparotomy revealed an extreme uterine torsion exceeding 360°, along with complete placental abruption and a Couvelaire uterus. Stepwise detorsion was necessary to restore proper uterine anatomy. After partial detorsion and identification of both adnexa, an anterior upper vertical uterine incision was made to enable delivery of the fetus and placenta. Hemorrhage was managed using uterotonics, tranexamic acid, fibrinogen concentrate, and temporary intrauterine tamponade. Despite the high bleeding risk, uterine preservation was successfully achieved. The patient recovered well postoperatively without significant complications. This case adds to the limited literature on severe uterine torsion and highlights the diagnostic and surgical challenges involved in high‐degree torsion complicated by placental abruption and Couvelaire uterus. It emphasizes the importance of anatomy‐guided intraoperative decision‐making and early, structured hemorrhage control. However, no definitive conclusions about the best surgical approach can be drawn from a single case.

## 1. Introduction

Uterine torsion, defined as a rotation of the uterus exceeding 45° on its longitudinal axis, is a rare obstetric emergency. Because clinical signs are often nonspecific, including abdominal pain, uterine hypertonus, or hemodynamic instability, the diagnosis is frequently made during cesarean delivery or laparotomy [1, 2]. When torsion is severe, distortion of uterine and pelvic anatomy can impair uteroplacental perfusion and venous outflow, increasing the risk of placental abruption, massive hemorrhage, and, in rare cases, Couvelaire uterus. High‐degree torsion complicates surgical orientation and uterine entry, raising the risk of hemorrhage and maternal morbidity. Published reports show wide variability in intraoperative management strategies, reflecting both the rarity of the condition and the lack of standardized surgical guidelines [3].

Adding to this complexity is the Couvelaire uterus (uteroplacental apoplexy), a rare complication of severe placental abruption where blood leaks into the myometrium, weakening uterine contractility and significantly increasing the risk of hemorrhage [1].

Recent case reports and narrative reviews have similarly emphasized the variability in torsion degree, operative approach, and maternal outcomes in uterine torsion, reflecting the absence of standardized management strategies [4].

A recent systematic review synthesized reported cases of uterine torsion and demonstrated marked heterogeneity in torsion degree, operative approaches, and maternal–fetal outcomes. Higher‐degree torsions (≥360°) were infrequently reported but were more frequently associated with placental abruption, fetal demise, and significant hemorrhage. These findings provide important context for extreme presentations such as the present case [5].

Here, we present a case of extreme uterine torsion complicated by placental abruption, Couvelaire uterus, and intrauterine fetal demise (IUFD), to highlight the diagnostic difficulty and intraoperative decision‐making challenges in such cases. This case is discussed within the context of the literature to illustrate management considerations, while recognizing the limitations inherent to single‐case reports.

## 2. Case Presentation

A 36‐year‐old Gravida 4 para 3 woman at 36+ weeks’ gestation presented at 06:52 AM with sudden, severe lower abdominal pain and dizziness. There was no vaginal bleeding. Her pregnancy had been uncomplicated except for a lower‐lying placenta noted at 26 weeks, which had resolved on follow‐up ultrasound at 35 weeks. She had no medical comorbidities and no history of abdominal surgery.

On admission, she was alert but drowsy. Her blood pressure was 90/50 mmHg, pulse 90 beats per minute, and oxygen saturation 99% on room air. Abdominal examination revealed a tense, tender uterus reaching the xiphisternum. Transabdominal ultrasound, obtained within ~10–15 min of presentation, confirmed IUFD with an upper‐lying placenta.

Vaginal examination showed no active bleeding, and cervical assessment was difficult due to distorted anatomy. Given persistent hypotension and clinical suspicion of placental abruption, the decision for emergency laparotomy was made within ~20 min of arrival. Skin incision occurred at ~07:15 AM, and fetal delivery was achieved at 07:25 AM.

Initial resuscitative measures included insertion of two large‐bore intravenous cannulas and collection of a blood for complete blood count: coagulation profile, including fibrinogen level; and crossmatch. A multidisciplinary team comprising senior obstetricians, anesthesiologists, intensivists, and radiologists was assembled.

### 2.1. Intraoperative Findings and Management

Laparotomy was performed via a Pfannenstiel incision. The uterus appeared markedly enlarged, congested, and bluish, consistent with a Couvelaire uterus.

**Figure 1:**
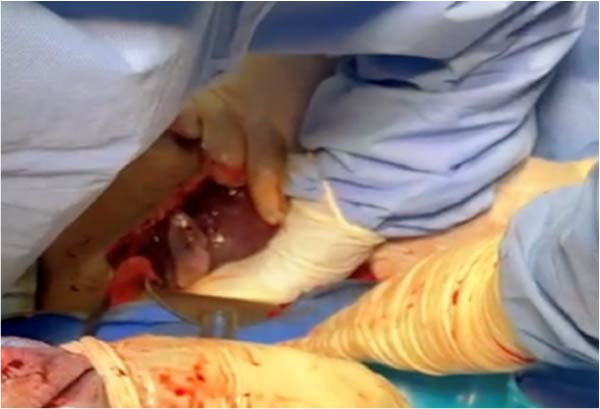
Intraoperative view at laparotomy demonstrating abnormal orientation of the uterus with displacement of the round ligaments and adnexa from their expected anatomical position, consistent with uterine torsion prior to complete anatomical correction (A picture showing the adnexa, ovary, at the abdominal incision site consists of more than 90° torsion).

**Figure 2:**
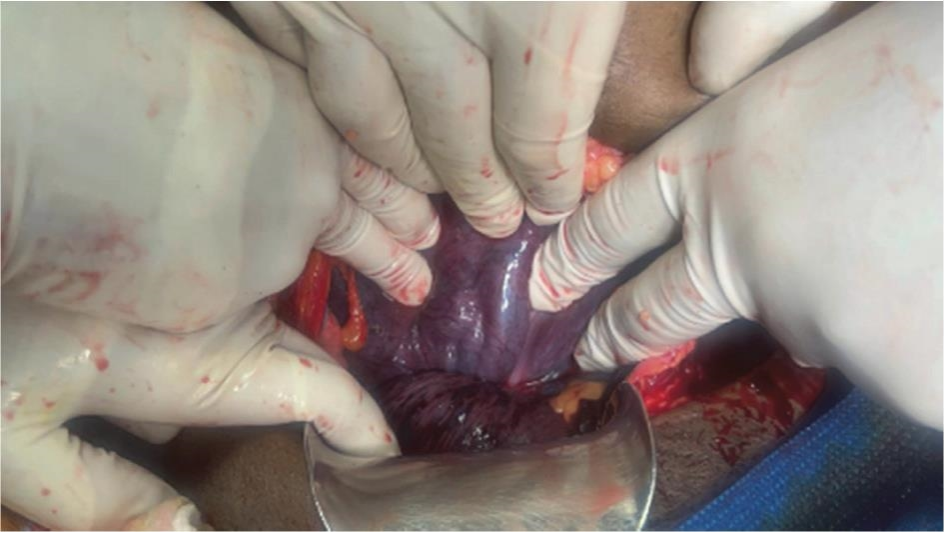
Intraoperative appearance of the gravid uterus showing marked congestion and bluish discoloration consistent with a Couvelaire uterus. A spiral (corkscrew) configuration of the lower uterine segment is visible, consistent with persistent uterine torsion prior to complete anatomical correction (Intraoperative photographs showing the grossly congested and couvelaire uterus with visible spiral rotation at the lower segment, corkscrew configuration, consistent with torsion of greater than 360°).

Intraoperative anatomical orientation was established through systematic identification of the round ligaments, fallopian tubes, and ovaries, which were displaced toward the right of midline, confirming rotational distortion. The spatial relationship between these structures and the lower uterine segment was carefully evaluated to determine the presumed anterior uterine surface before incision (Figure 1). A spiral configuration at the level of the lower uterine segment (Figure 2) suggested persistent torsion, and despite landmark recognition, exact anterior–posterior orientation remained partially uncertain.

Manual detorsion was initially performed to reorient the uterus and facilitate safer uterine entry partially. After partial correction, an anterior upper vertical uterine incision was made. The fetus was delivered in cephalic presentation at 07:25 AM, followed by expulsion of the placenta and ~1 liter of retroplacental clot, confirming complete placental abruption.

**Figure 3:**
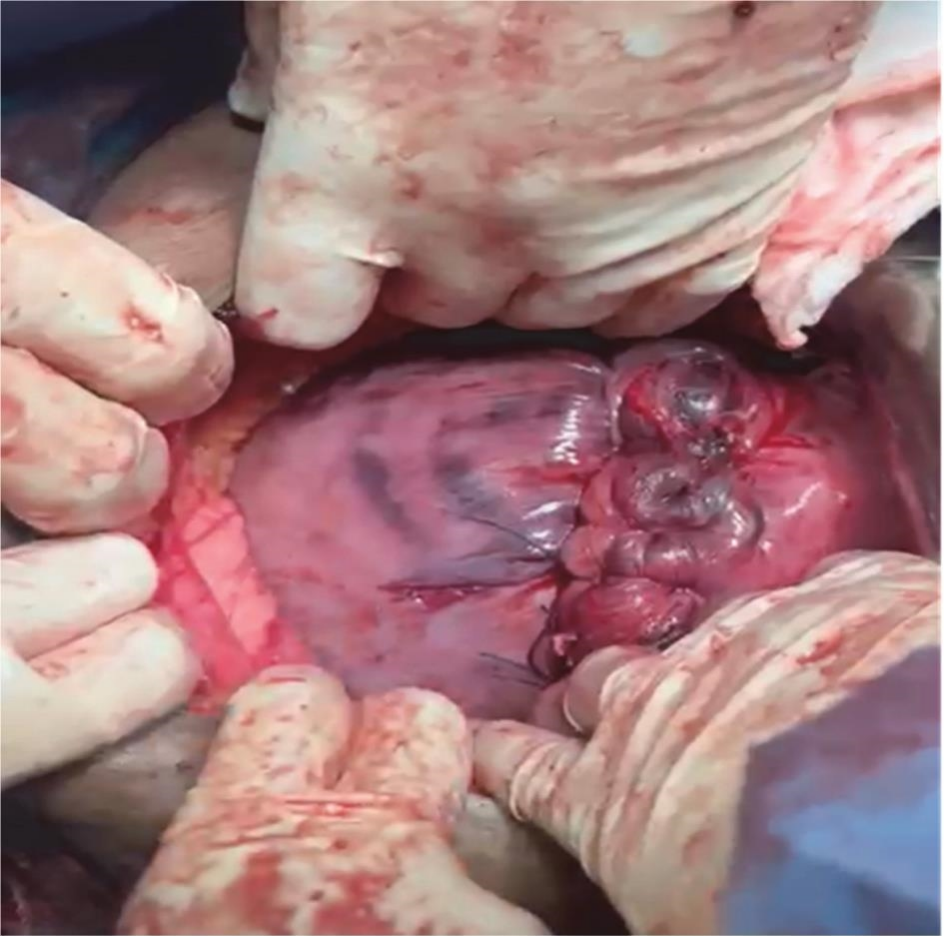
Exteriorized uterus following delivery and sequential detorsion maneuvers, demonstrating restoration of normal uterine orientation and improved coloration after complete anatomical correction (This figure shows the uterus after detorsion and delivery of the fetus; it has regained a pink color, and the C‐section scar is visible).

Following delivery, the uterus was exteriorized for reassessment. Persistent torsion was identified, requiring three additional sequential detorsion maneuvers to restore normal uterine anatomy fully. After complete correction, the uterine coloration and appearance improved (Figure 3). The need for sequential detorsions before and after delivery suggested a high‐degree uterine torsion.

### 2.2. Hemorrhage Management

Intraoperative bleeding was brisk. Temporary intrauterine packing with abdominal towels was applied to achieve hemostasis. Uterotonic agents, including oxytocin, methylergometrine, and carboprost, were administered, along with tranexamic acid and 2 g of fibrinogen concentrate.

The estimated intraoperative blood loss was ~2000 mL. The patient received a total of five units of packed red blood cells and two units of fresh frozen plasma during her hospitalization. Transfusion and fibrinogen supplementation were guided by serial laboratory assessment and institutional obstetric hemorrhage protocols. Massive transfusion protocol was not formally activated, as hemodynamic stability improved with targeted, laboratory‐guided transfusion. Transient vasopressor support with norepinephrine was required intraoperatively.

Once hemostasis was secured, the uterine incision was closed in layers, and an abdominal drain was inserted.

### 2.3. Postoperative Course

Postoperatively, the patient was transferred to the intensive care unit for close monitoring. Initial laboratory evaluation revealed a hemoglobin of 6.5 g/dL, with normal platelet count and coagulation parameters.

Over the following 24 h, uterine tone remained adequate with minimal vaginal bleeding and stable drain output. Additional transfusion support was administered as required. Prophylactic low‐molecular‐weight heparin was initiated once the bleeding risk had decreased.

The patient remained hemodynamically stable and was discharged from the ICU on postoperative day 2 and from the hospital on postoperative day 4. She recovered without further complications.

## 3. Discussion

Uterine torsion is a rare but potentially catastrophic obstetric emergency that is seldom diagnosed before surgery because of its nonspecific and often misleading clinical signs. Patients may present with sudden abdominal pain, uterine hypertonus, or hemodynamic instability, often without vaginal bleeding, which limits the effectiveness of clinical assessment and imaging. As a result, uterine torsion is most often identified during surgery, such as laparotomy or cesarean delivery, as seen in our case [1–3].

### 3.1. Spectrum of Uterine Torsion and Positioning of the Present Case

Most reported cases of uterine torsion involve rotations of 180° or less and generally have good maternal and fetal outcomes when treated promptly. These lower‐degree torsions often allow safe uterine entry after minimal or no detorsion and rarely lead to severe bleeding or loss of the uterus. Table 1 summarizes previous cases within this range, providing useful clinical context [6–10].

**Table 1:** Reported cases of lower‐degree uterine torsion (≤180°): clinical presentation, operative approach, and maternal–fetal outcomes.

Author (Year)	Journal	Gestational age	Degree of torsion	Clinical presentation	Uterine incision	Fetal outcome	Maternal outcome
Picone et al. [6]	Obstetrics & Gynecology	Term	180°	Acute abdominal pain	Posterior hysterotomy	Live birth	Uneventful recovery
Wilson et al. [7]	Journal of Obstetrics and Gynaecology Canada	28–36 weeks	~90°–180°	Abdominal pain	Classical incision	Live birth	Uterus preserved
Zullino et al. [8]	Case Reports in Obstetrics and Gynecology	33 weeks	180°	Pain, placental abruption	Posterior incision	Live birth	Uneventful recovery
Jones et al. [9]	International Journal of Reproduction, Contraception, Obstetrics and Gynecology	35 weeks	180°	Abdominal pain	Posterior incision	Live birth	Uneventful recovery
Lawrence et al. [10]	Cureus	Term	≥45°–180°	Abdominal pain	Variable	Mostly live births	Variable

*Note:* This table summarizes published cases of uterine torsion with rotation ≤180°, highlighting gestational age at presentation, degree of torsion, surgical approach, and outcomes. These cases provide baseline clinical context and are generally associated with favorable maternal and fetal outcomes when timely intervention is undertaken. References supporting individual cases are summarized within the table and are not repeated in the main text.

This case represents the extreme end of the torsion spectrum and is best classified as higher‐degree uterine torsion, likely exceeding 360°. This is supported by intraoperative findings: significant spiral distortion of the lower uterine segment, abnormal displacement and positioning of the adnexa, and the need for multiple detorsion steps after delivery before normal uterine anatomy was restored. These features are typical of extreme uterine torsion, as described in the literature, but are rarely reported [4, 5, 11].

**Figure 4:**
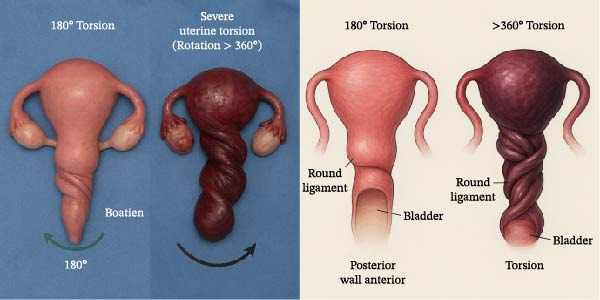
Schematic illustration comparing lower‐degree (≤180°) and higher‐degree (≥360°) uterine torsion, demonstrating progressive spiral distortion of the lower uterine segment, round ligaments, and adnexa. This AI‐generated illustration was created as a conceptual visualization based on published literature and intraoperative findings and is intended to aid anatomical interpretation rather than serve as a diagnostic image (This is an AI‐generated image based on the analysis of all the mentioned case series data, along with a description of our case to illustrate the difference between uterine torsion <180° and 360°, and to confirm the diagnosis of 450° in our case based on the image).

To aid interpretation of the anatomy, a schematic diagram (Figure 4) illustrates the intraoperative configuration. Created using AI, it aims to contrast lower‐ and higher‐degree uterine torsion based on published research and surgical observations. This figure is meant as an aid for understanding rather than a diagnostic tool, supporting the classification of this case within the high‐degree torsion category.

High‐degree uterine torsion is more often linked to poor venous drainage and reduced uteroplacental blood flow, increasing the risk of placental abruption, fetal death, and severe maternal bleeding [4, 5]. All these complications occurred in this case, highlighting the seriousness of the presentation.

### 3.2. Intraoperative Orientation, Detorsion, and Surgical Decision‐Making

Severe uterine torsion causes major distortion of pelvic anatomy, making intraoperative orientation and safe uterine access difficult. In this case, an initial partial detorsion was deliberately performed because it was feasible and only enough to identify the adnexa and safely make the uterine incision. Full anatomical correction was achieved only after delivery and exteriorization of the uterus, when persistent torsion was evident, requiring additional detorsion steps.

This approach emphasizes an important point: In suspected high‐degree torsion, the full extent of rotation may not be clear before delivery, and repeated reassessment of anatomy is crucial. Similar findings have been reported, showing that uterine orientation can change during surgery and that reevaluation after delivery may reveal residual torsion that was not initially obvious [3, 4, 11].

The literature describes various surgical strategies for similar cases, including posterior uterine incisions when detorsion is not possible. These methods are often used in cases of extreme anatomical distortion and uncontrolled bleeding and have been linked to higher hysterectomy rates [4, 5]. However, these reports are descriptive, and no standard surgical technique exists. The approach used here should be viewed as a practical option tailored to intraoperative findings and maternal condition, not a universally applicable or superior technique.

### 3.3. Hemorrhage, Couvelaire Uterus, and Maternal Outcome

High‐degree uterine torsion can impair uteroplacental blood flow and venous drainage, increasing the risk of placental abruption and leading to a Couvelaire uterus. This condition indicates extensive bleeding within the myometrium and is associated with uterine atony and postpartum hemorrhage [1, 12]. In this case, early preparation for bleeding complications guided the use of uterotonics, tranexamic acid, fibrinogen supplements, and temporary intrauterine tamponade, following strategies from previous reports [1, 5].

Despite the severe uterine torsion, complete placental abruption, and presence of a Couvelaire uterus, the uterus was preserved, and the mother recovered without complications. This positive outcome was likely due to prompt intervention, repeated anatomical reassessment, and coordinated bleeding control, rather than any single surgical maneuver.

### 3.4. Clinical Decision‐Making, Team Coordination, and Response Time

In rare obstetric emergencies like uterine torsion, maternal outcomes depend on more than just surgical findings—they rely heavily on timely, coordinated clinical action. In this case, early recognition of maternal instability, quick confirmation of fetal demise, and rapid involvement of senior obstetric, anesthesia, intensive care, and radiology teams enabled expedited operative management in a controlled surgery [1, 3].

Avoiding prolonged diagnostic delays, initiating early resuscitation, preparing blood products, and planning anesthesia collectively enabled an organized response to a life‐threatening problem. This multidisciplinary approach enabled continuous reassessment of anatomy and stability, especially given the growing recognition of persistent high‐degree torsion after delivery and exteriorization of the uterus. Although the exact impact of response time cannot be measured here, this case illustrates that early team involvement and prompt intraoperative reassessment can support maternal stabilization in cases of extreme uterine torsion.

### 3.5. Comparison With Published Literature and Limitations

Cases of uterine torsion over 360° are rare, and many involve severe bleeding, hysterectomy, or maternal complications [3–5, 11]. Table 2 summarizes previous high‐degree torsion cases, while Table 3 compares low‐ and high‐degree torsion with respect to presentation, surgical strategies, and outcomes. These comparisons reveal the wide variation and absence of standardized procedures.

**Table 2:** Reported cases of higher‐degree uterine torsion (≥360°) complicated by placental abruption and/or intrauterine fetal demise.

References	Journal	Gestational age	Degree of torsion	Associated complications	Operative findings	Surgical management	Maternal outcome
Kilicci et al. [12]	Pan African Medical Journal	34 weeks	720°	Bladder torsion, IUFD	Severe anatomical distortion	Detorsion + cesarean	Uterus preserved
Protrka et al. [11]	Clinical and Experimental Obstetrics & Gynecology	Term	≥360°	Abruption, IUFD	Couvelaire uterus	Cesarean + hysterectomy	Survived
Kumar et al. [4]	Journal of Obstetrics and Gynaecology	Third trimester	≥360°	IUGR/fetal compromise	Marked torsion	Cesarean	Variable
Jensen [13]	Acta Obstetricia et Gynecologica Scandinavica	Term	≥360°	Abruption	Myometrial hemorrhage	Cesarean	Uterus preserved
Present case	—	36 weeks	≥360° (inferred)	Abruption, IUFD, Couvelaire uterus	Spiral distortion adnexal displacement	Stepwise detorsion + cesarean	Uterus preserved

*Note:* This table provides a comparative summary of published cases of high‐degree uterine torsion associated with severe obstetric complications, including placental abruption, Couvelaire uterus, and adverse fetal outcomes. Degree of torsion is frequently inferred intraoperatively based on anatomical configuration and detorsion requirements rather than direct measurement. References are incorporated within the table to avoid duplication in the discussion.

**Table 3:** Comparison between lower‐degree (≤180°) and higher‐degree (≥360°) uterine torsion based on published reports.

Feature	Lower‐degree torsion (≤180°)	Higher‐degree torsion (≥360°)	Key references ^∗^
Frequency in literature	More commonly reported	Rarely reported	[3, 4]
Typical presentation	Abdominal pain ± labor	Abdominal pain, shock, collapse	[3, 4]
Preoperative diagnosis	Rare	Extremely rare	[3]
Anatomical distortion	Mild to moderate	Severe spiral distortion	[5, 11]
Adnexal orientation	Usually identifiable	Markedly displaced	[4, 5]
Placental abruption	Uncommon	Commonly reported	[3–5, 11]
Couvelaire uterus	Rare	Frequently reported	[5, 12]
Fetal outcome	Usually live birth	High rate of IUFD	[3–5, 11]
Uterine incision	Often posterior or classical	Variable, detorsion often required	[2–5]
Detorsion required	Minimal or none	Often multiple, sequential	[4, 5]
Hemorrhage risk	Low to moderate	High	[1, 5, 12]
Need for hysterectomy	Rare	Frequently reported	[5, 11]
Maternal outcome	Generally favorable	Variable, increased morbidity	[2–5, 11]
Evidence base	Case reports, small series	Isolated case reports	[2–5, 11]
Strength of evidence	Descriptive	Descriptive	[2–5, 11]

*Note:* This table contrasts key clinical features, intraoperative findings, and outcomes between lower‐ and higher‐degree uterine torsion as reported in the literature. The comparison is descriptive and derived from case reports and small case series. Key references supporting each comparative feature are provided within the table.

This report presents a detailed case of high‐grade uterine torsion, supported by intraoperative observations, structured treatment, and schematic anatomy. However, conclusions from a single case should be cautious, and management should always be tailored to the specific intraoperative situation and the stability of the maternal condition.

## 4. Conclusion

Extreme uterine torsion complicated by placental abruption and Couvelaire’s uterus is a rare yet life‐threatening obstetric emergency. This case demonstrates that prompt detorsion, anatomy‐guided uterine entry, and early, structured hemorrhage control can be associated with successful uterine preservation, even when IUFD occurs. However, management decisions must be individualized, and no causal conclusions can be drawn from a single case. Multidisciplinary preparedness and intraoperative flexibility are essential to optimize maternal outcomes in such situations.

## Author Contributions

The author confirms sole responsibility for the conception, design, data collection, analysis, interpretation, manuscript drafting, and final approval of the version to be published.

## Funding

No funding was received for the preparation of this manuscript.

## Ethics Statement

Ethical approval was not required for this case report, according to the maternity hospital’s policies, as it describes a single anonymized patient. Written informed consent was obtained from the patient for publication of this report and any accompanying images.

## Conflicts of Interest

The author declares no conflicts of interest.

## Data Availability

Data sharing not applicable to this article as no datasets were generated or analyzed during the current study.
